# Finite Element Modeling of Debonding Failures in FRP-Strengthened Concrete Beams Using Cohesive Zone Model

**DOI:** 10.3390/polym14091889

**Published:** 2022-05-05

**Authors:** Mohammed A. Al-Saawani, Abdulaziz I. Al-Negheimish, Ahmed K. El-Sayed, Abdulrahman M. Alhozaimy

**Affiliations:** Center of Excellence for Concrete Research and Testing, Department of Civil Engineering, King Saud University, P.O. Box 800, Riyadh 11421, Saudi Arabia; malsaawani@ksu.edu.sa (M.A.A.-S.); negaimsh@ksu.edu.sa (A.I.A.-N.); alhozimy@ksu.edu.sa (A.M.A.)

**Keywords:** concrete beams, FRP strengthening, IC debonding, concrete cover separation, cohesive zone model, shear span-to-depth ratio

## Abstract

Intermediate crack (IC) debonding and concrete cover separation (CCS) are common types of debonding failures in concrete beams flexurally strengthened with fiber-reinforced polymer (FRP) composites. In this paper, a three-dimensional finite element (FE) model was developed to simulate the flexural behavior and predict the critical debonding failure in FRP-strengthened beams. The two critical debonding failures were considered in the FE model by implementing a cohesive zone model based on fracture mechanics considering the effect of the related parameters. The input values used for the cohesive zone model are modified in this study to obtain accurate and consistent predictions. The FE model was validated by comparison with experimental results tested by the authors for beams particularly prone to fail by either of the two critical debonding failures. The results obtained from the FE model agree well with the experimental results for both of the debonding failures and the corresponding capacities at failure. In general, the ratio of the experimental to numerical ultimate capacities was within 5%, and so was the ratio of the experimental to numerical mid-span deflections at debonding failures. The FE model developed in this study was then used to conduct a parametric study investigating the effect of shear span-to-depth ratio and spacing of steel stirrups on the ultimate capacity and type of debonding failure in FRP-strengthened beams. The results of the parametric study revealed that increasing the spacing of steel stirrups caused a significant decrease in the load capacity at concrete cover separation failure. In addition, varying the shear span-to-depth ratio was seen to have an important effect on the type of debonding failure and corresponding capacities for the FRP-strengthened beams having the same cross-section geometry and CFRP reinforcement.

## 1. Introduction

Fiber-reinforced polymer (FRP) is a composite material made of high-tensile-strength fibers glued by a polymer matrix. These fibers are usually made of carbon, glass, or aramid. The polymer used is usually an epoxy, vinylester, or polyester thermosetting plastic. FRP composites are lightweight, noncorrosive, easily constructed; higher in tensile strength; and can be applied to achieve the performance requirements. In particular, carbon FRP (CFRP) has excellent mechanical and fatigue properties compared to other types of FRP composites [1]. Due to these characteristics, FRP composites have gained wide acceptance and utilization in the rehabilitation of concrete structures.

**Figure 1:**
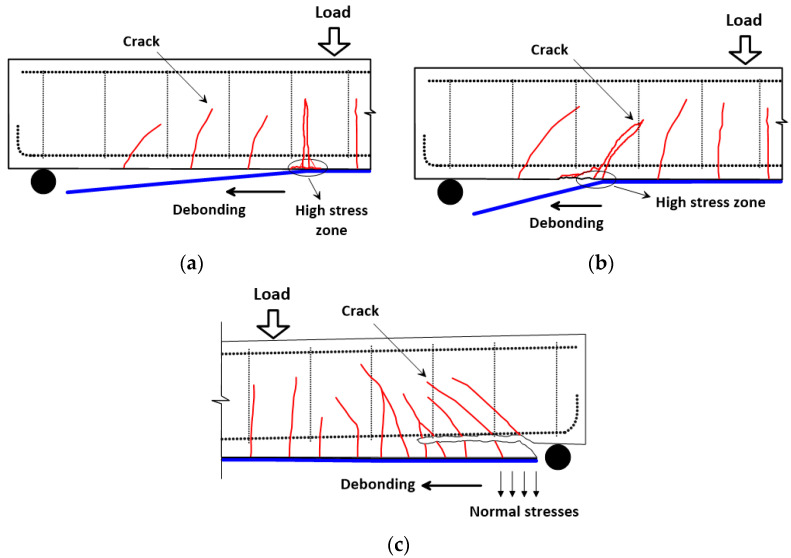
Common types of debonding failures in FRP-strengthened beams: (**a**) interfacial debonding induced by intermediate flexural cracks; (**b**) interfacial debonding induced by intermediate flexural-shear cracks; (**c**) concrete cover separation.

Strengthening of reinforced concrete (RC) members using FRP externally bonded (EB) to the concrete substrate is an excellent method to increase the load capacity of such members. Many experimental and analytical investigations have been conducted and their results have indicated the effectiveness of FRP strengthening in increasing the load-carrying capacity of RC members [2,3,4,5,6,7,8]. However, these studies have reported that only a small percentage of FRP tensile strength is utilized before the premature failure by FRP debonding. The most common debonding failures in FRP-strengthened beams are intermediate crack (IC)-induced interfacial debonding (IC debonding) and concrete cover separation (CCS) failure at the plate-end region. Figure 1 schematically shows these two common types of debonding failure modes in FRP-strengthened beams. The IC debonding failure could initiate at the toe of a flexural crack in the high-moment region at the middle of the beam, and then propagate along the direction of decreasing moment towards the FRP plate end, as shown in Figure 1a. The IC debonding failure may also initiate at the toe of a flexural-shear crack in the shear span, and then propagate towards the FRP plate end, as shown in Figure 1b. On the other hand, CCS failure, being the most common failure type that occurs at the plate-end region, initiates because of the combined interfacial shear and normal stresses that are generated at the FRP plate end, resulting in the formation of splitting cracks in the concrete. The cracks propagate from the bottom of the beam to the level of the tension steel, and then propagate horizontally onward along the level of the tension steel, eventually causing the separation of the concrete cover, as shown in Figure 1c.

Many factors can affect the occurrence of a particular debonding failure mode, including the concrete cover thickness; the number and size of tension steel bars; the distance between FRP plate end and the beam support; FRP plate length, width, thickness, and modulus of elasticity; shear span-to-depth ratio; shear-to-moment interaction; section geometry; and concrete strength [6,9,10,11]. Among the various factors that affect the critical debonding failure in FRP-strengthened beams, Al-Saawani et al. [6] have conducted an experimental study that revealed the significant effect of shear span-to-depth ratio on the controlling debonding failure mode.

The finite element (FE) method is a useful tool for improving the understanding of the behavior of RC beams strengthened with FRP composites. Many FE models have been developed for simulating the behavior of FRP-strengthened RC beams in which different approaches have been considered. In some FE studies, a full-bond assumption was considered between the FRP and concrete substrate in the simulation of the response of the strengthened members [12,13,14]. Other numerical studies have considered modeling the adhesive layer as a linear-elastic material [15,16]. To better describe the behavior at the interface between the FRP reinforcement and concrete, other FE studies used interface elements [17,18,19]. Among available bond-slip models, Lu et al.’s model [20] has received the most acceptance for describing the bond behavior of FRP-to-concrete interfaces [21,22,23,24]. Pham and Al-Mahaidi [25] used a bilinear model for the FRP-to-concrete interface. The parameters of the adopted bilinear bond-slip model were directly determined from tests conducted on bonded joints.

Numerical studies using FE modeling are used for investigating debonding failures in FRP-strengthened members. Modeling of damage using FE analysis can be generally classified as discrete or continuous [26]. The two common modeling approaches are the fracture-energy-based cohesive zone model (CZM) and the continuum damage mechanics (CDM) approach. In the CZM approach, the adhesive is represented using interface elements in which their behavior is characterized using a traction-separation law. The CDM approach, on the other hand, is based on the stiffness degradation of the adhesive elements as imposed by a damage parameter. Both modeling approaches are able to predict both damage initiation and propagation. CZM can be used to represent delamination, whereas CDM accounts for matrix and fiber damage [26]. The increasing use of the CZM approach is attributed to its ability to simulate the initiation and progression of the debonding failure, and because of its ease of implementation, as it has been included as a built-in feature in many commercial FE software packages [27].

The cohesive zone model (CZM) is a recently applied method in the simulation of composite materials. This model assumes that the stress transfer capacity between the two separating faces of delamination is not completely lost at damage initiation [28]. It is rather a progressive event as governed by progressive stiffness reduction of the interface between the two separating faces. Existing FE models used for the prediction of IC debonding in FRP-strengthened beams based on the CZM derive the interface shear stress distribution law by defining a nonlinear interface bond-slip relationship [29,30,31]. This indicates the important role of the bond slip curve that is considered in the prediction of IC debonding failure. The constitutive relation of the FRP–concrete interface is mostly described by a bilinear model because of its simplicity and accurate prediction of the interface debonding [22,32,33]. Zidani et al. [30] presented an FE model to simulate the flexural behavior of initially damaged concrete beams repaired with FRP plates. The model used the bond stress-slip model proposed by Lu et al. [20] to characterize the interface elements between the EB FRP and concrete. Zhang et al. [34] proposed an FE model for the prediction of IC debonding in FRP-strengthened RC beams based on fracture mechanics and the cohesive zone model. The constructed model also relied on theoretical derivations and available experiments.

Unlike FE modeling of IC debonding failure, numerical FE studies on beams failing by CCS failure are limited. Supaviriyakit et al. [35] used the smeared crack model to analyze FRP-strengthened RC beams. In their analysis, the FRP-to-concrete interface was assumed to be perfectly bonded, and the steel bars were uniformly distributed in the concrete elements without any additional nodes and elements. The model predictions showed close agreement with the test results; however, the predicted crack pattern was unclear. Pham and Al-Mahaidi [25] proposed an FE approach using a rotating smeared crack model for the concrete part under the level of tension steel, while a fixed smeared crack model was used for the concrete part above it. A perfect bond was assumed for the modeling of the steel-to-concrete interface. For modeling the FRP-to-concrete interface, a bilinear model was used, and the parameters considered in the bond-slip model were determined from shear tests of bonded joints. A more advanced model was proposed by Zhang and Teng [36], in which a two-dimensional FE approach was used for the prediction of end cover separation failure in concrete beams flexurally strengthened with FRP. The proposed approach considered the cracking of concrete, the bond behavior between steel bars and concrete and between FRP and concrete, and the radial stresses exerted by tension steel bars onto the surrounding concrete. Sakr [37] constructed FE models to analyze continuous beams strengthened with CFRP composites. The study took the two debonding failure modes into account by using cohesive surfaces. In this model, the relation between the concrete and the steel reinforcement was assumed to be perfectly bonded, as modeled by the embedded region constraint. However, no description of the cohesive zone model used for modeling CCS failure was provided. In addition, a clear presentation for the type of debonding failure was lacking.

The conducted literature review indicates that most of the available numerical studies considered one of the debonding failure modes in the simulation of FRP-strengthened beams. The current numerical study is directed to consider both IC debonding and CCS failures in FRP-strengthened beams. In this paper, the development of a three-dimensional FE model using the commercial FE software ABAQUS [38] is presented. The FE model is developed to simulate the flexural behavior of FRP-strengthened RC beams and predict the controlling debonding failure mode. Nonlinear relations for the constituent materials are considered in this model. In addition, the interfacial shear and normal stresses at the level of the adhesive layer and also those at the level between the tension steel bars and concrete cover are considered using CZM based on nonlinear fracture mechanics. The FE model is validated using experimental data of beams previously tested by the authors, and then used to investigate the effect of shear span-to-depth ratio and spacing of steel stirrups on the behavior and mode of debonding failure in FRP-strengthened beams.

## 2. Development of FE Model

A three-dimensional nonlinear FE model for simulating the flexural behavior of FRP-strengthened RC beams is developed in this study using the FE package ABAQUS [38]. Only one quarter of the FRP-strengthened beam was modeled in order to reduce the calculation time, and the FE results for the whole beam could be derived using the principle of symmetry.

### 2.1. Material Properties and Constitutive Models

#### 2.1.1. Concrete

Among the available approaches in ABAQUS to simulate the behavior of concrete, the concrete damaged plasticity (CDP) model was used in this study. Such model requires the values of elastic modulus, Poisson’s ratio, plastic damage parameters, and description of compressive and tensile behavior. The plastic damage parameters include the dilation angle (*Ψ*), the flow potential eccentricity (*ϵ*), the ratio of initial biaxial compressive yield stress to initial uniaxial compressive yield stress (*f_b_*_0_/*f_c_*_0_), the ratio of the second stress invariant on the tensile meridian to that on the compressive meridian (*K*), and the viscosity parameter that defines visco-plastic regularization. The values of plastic damage parameters are shown in Table 1 as recommended by ABAQUS documentation for the definition of concrete material. The Poisson’s ratio of concrete was chosen to be 0.2.

To model the behavior of concrete in compression, the stress-strain curve of concrete for a given concrete characteristic compressive strength can be described using a suitable model, such as the one developed by Carreira and Chu [39], as follows:

(1) $$f_{c}\;=\;\frac{f_{c}^{\prime }\beta \left(\varepsilon /\varepsilon_{0}\right)}{\beta \;-\;1\;+\;{\left(\varepsilon /\varepsilon_{0}\right)}^{\beta }}$$

(2) $$\beta \;=\;\frac{1}{1\;-\;\left(\frac{f_{c}^{\prime }}{E_{0}\varepsilon_{0}}\right)}$$

(3) $$E_{0}\;=\;\frac{f_{c}^{\prime }}{\varepsilon_{0}}\left(\frac{24.82}{f_{c}^{\prime }}\;+\;0.92\right)$$

(4) $$\varepsilon_{0}\;=\;\left(1680\;+\;7.1f_{c}^{\prime }\right)\;\times \;10^{-6}$$

where *f_c_* is the concrete stress; *f*′*_c_* is the maximum stress; *β* is a material parameter; *ε* is the concrete strain; *ε_0_* is the corresponding strain at maximum stress; and *E*_0_ is the initial tangent modulus of elasticity.

**Figure 2:**
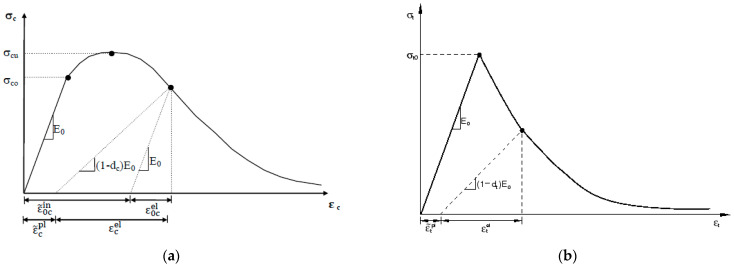
Response of concrete to uniaxial loading [38]: (**a**) response of concrete in compression; (**b**) response of concrete in tension.

The stress-strain curve can be defined beyond the ultimate stress into the strain-softening regime. The compressive inelastic strain, ${\tilde{\varepsilon }}_{0c}^{in}$, is defined as the total strain minus the elastic strain, ${\tilde{\varepsilon }}_{0c}^{in}\;=\;\varepsilon_{c}\;-\;\varepsilon_{0c}^{el}$, as illustrated in Figure 2a.

The concrete behavior in tension is modeled using a linear elastic approach until cracking is initiated at its tensile strength. After crack initiation, the softening starts, and the post-failure behavior for direct straining is modeled with tension stiffening, which allows one to define the strain-softening behavior for cracked concrete. It is possible to specify tension stiffening by means of a post-failure stress-strain relation or by applying a fracture energy cracking criterion. The response of concrete to uniaxial loading in tension is shown in Figure 2b.

For concrete under uniaxial tension, Hordijk [40] proposed the following tension-softening curve based on an extensive series of tension tests of concrete. This model is also considered in this study and is as follows:

(5) $$\sigma_{t}\;=\;f_{ct}\left[1\;+\;{\left(c_{1}\frac{w_{t}}{w_{cr}}\right)}^{3}\right]e^{\left(-c_{2}\frac{w_{t}}{w_{cr}}\right)}\;-\;\frac{w_{t}}{w_{cr}}\left(1\;+\;c_{1}^{3}\right)e^{\left(-c_{2}\right)}$$

(6) $$w_{t}\;=\;\varepsilon_{cr}h_{c}$$

(7) $$\varepsilon_{cr}\;=\;\varepsilon_{t}\;-\;\varepsilon_{e}$$

(8) $$w_{cr}\;=\;5.14\frac{G_{F}}{f_{ct}}$$

where *w_t_* and *ε_cr_* are the crack opening displacement and cracking strain, respectively; *ε_t_* and *ε_e_* are the total strain and elastic tensile strain, respectively; *w_cr_* is crack opening displacement at the complete release of stress or fracture energy; *σ_t_* is tensile stress normal to the crack direction; *f_ct_* is concrete uniaxial tensile strength; *G_F_* is fracture energy required to create a stress-free crack over a unit area; and *c*_1_ = 3.0 and *c*_2_ = 6.93 are constants determined from tensile tests of concrete. In FE simulations, *f_ct_* could be calculated by ACI 318 [41], and *G_F_* was considered from CEB-FIP [42] as follows:

(9) $$f_{ct}\;=\;0.33\sqrt{f_{c}^{\prime }}$$

(10) $$G_{F}\;=\;\left(0.0469d_{a}^{2}\;-\;0.5d_{a}\;+\;26\right){\left(\frac{f_{c}^{\prime }}{10}\right)}^{0.7}$$

where *d_a_* (in mm) is the maximum aggregate size, and *f*′*_c_* is the cylinder compressive strength (in MPa).

The stress-displacement curve defined by Equations (5)–(8) can be transformed into a stress-strain curve according to the crack band model. In ABAQUS [38], the crack band width *h_c_* is defined as the characteristic crack length of an element. In this study, Rots’ [43] recommendation for estimating the crack band width is followed. For instance, the characteristic crack length of a plane stress four-node square element with four integration points is taken to be $\sqrt{2}e$, where *e* is the side length of the element.

#### 2.1.2. Steel Reinforcement

**Figure 3:**
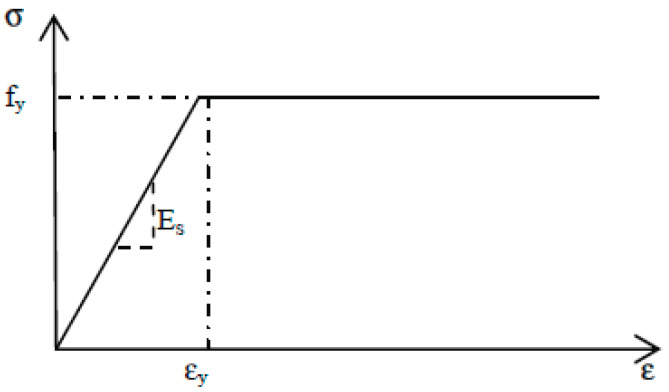
Stress-strain behavior of steel reinforcement.

The steel reinforcements were modeled as an elastic perfectly plastic material, as shown in Figure 3. The input for the steel model includes elastic modulus, Poisson’s ratio, and yield stress.

#### 2.1.3. FRP Reinforcement

The CFRP laminate was modeled as a linear elastic behavior up to the brittle failure, where the CFRP composite is mainly stressed in the fiber direction. The elastic behavior was modeled as a lamina type in ABAQUS. The mechanical properties of the CFRP lamina include the ultimate tensile strength, *f_u_*, Poisson’s ratio, *ν*, and the Young’s modulus and shear modulus, *E* and *G*, that are associated with the material’s principal directions “1, 2, 3”, which represent the longitudinal, transversal, and normal directions, respectively.

### 2.2. Modeling of Debonding Failures

FRP-strengthened beam could fail by two different debonding modes, either by interfacial debonding of FRP within a thin layer of concrete (IC debonding) or by the separation of concrete cover due to a combination of interfacial shear and normal stresses concentrating near the plate-end region. Therefore, two different failure modes were considered in the proposed FE model. This FE study takes the two possible types of debonding failure into account by considering two cohesive surfaces. The first cohesive surface is considered at the level of the adhesive layer to account for IC debonding failure, whereas the second cohesive surface is inserted between the tension steel bars and the concrete cover to account for CCS failure. Properties of both the adhesive and concrete are considered for the two cohesive surfaces.

#### 2.2.1. Modeling of IC Debonding

Using a perfect bond between CFRP and concrete results in overestimated predictions of the load capacity and stiffness compared to experimental results. Therefore, for IC debonding failure, the interface between CFRP laminate and concrete substrate needs to be modeled with an appropriate interaction. The cohesive model available in ABAQUS is a suitable choice for representing such interface behavior. The failure of the cohesive bond is characterized by progressive degradation of the cohesive stiffness, which is driven by a damage process. For this purpose, eight-node 3D cohesive elements (element COH3D8 in ABAQUS) were used to model the interface layer based on a proper bond-slip model.

**Figure 4:**
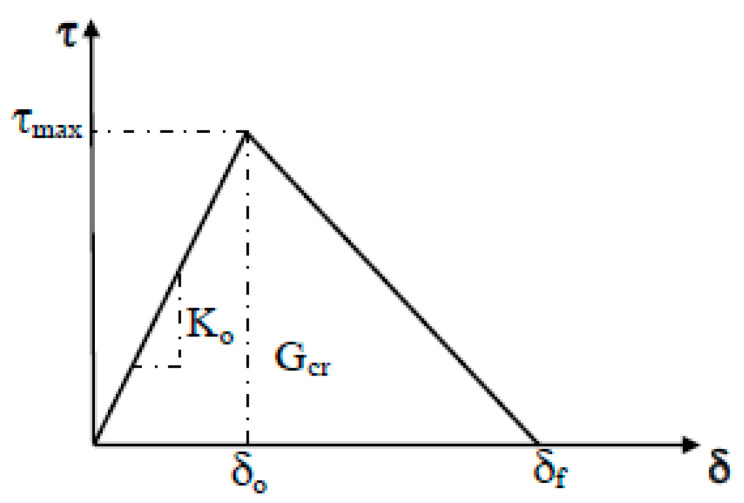
Bilinear bond-slip curve.

The definition of the cohesive zone model is characterized by parameters of initial stiffness, shear strength, fracture energy, and curve shape of the bond-slip model. In this study, the constitutive relation of the FRP–concrete interface is described by a bilinear model. Such a model is widely used to define the interface behavior of FRP-strengthened RC beams because it is convenient for use and gives accurate prediction of interface debonding [32,33]. A graphical representation of a bilinear traction-separation law is depicted in Figure 4. It can be observed from Figure 4 that the traction-separation law is defined by three parameters: initial stiffness (*K_o_*), normal (*σ_max_*) or shear bond strength (*τ_max_*), and fracture energy (*G_cr_*), which is equal to the area under the traction-displacement curve. The nominal traction stress vector consists of three components, *σ_n_*, *τ_t_*, and *τ_s_*, which represent the normal and shear tractions, respectively. The constitutive equations for the bond-slip law are as follows:

(11) $$\tau \;=\;\left\{\begin{array}{c} \frac{\tau_{max}}{\delta_{o}}\delta \;0\;\leq \;\delta \;\leq \;\delta_{o}\\ \frac{\tau_{max}}{\delta_{f}\;-\;\delta_{o}}\left(\delta_{f}-\delta \right)\;\delta_{o}\leq \delta \leq \delta_{f}\\ 0\;\delta >\delta_{f} \end{array}\right.$$

where *τ_max_* is the shear strength of the interface, and 𝛿*_f_* is the bond separation slip when the interfacial shear stress is reduced to zero. The area surrounded by the bilinear curve represents the interface fracture energy *G_cr_*, which can be calculated as *G_cr_* = ½*τ_max_*𝛿*_f_*.

For modeling the behavior of the FRP-to-concrete interface, the bond-slip interface model proposed by Lu et al. [20] was used. The considered interface model is based on traction-separation laws (cohesive behaviors). The equations of the bond–slip law model used in this study are summarized in Table 2, in which *b_f_* and *b* represent the width of the CFRP laminate and concrete beam, respectively. The tensile strength of concrete, *f_ct_*, expressed in MPa, is calculated by Equation (9).

**Figure 5:**
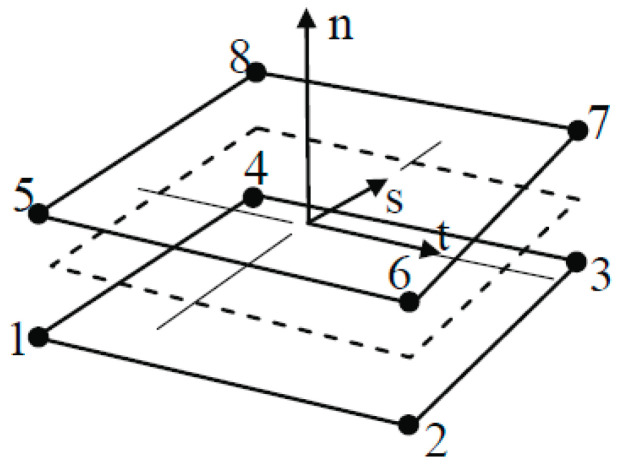
Eight-node 3D cohesive element [38].

The initiation of damage is assumed to occur when a quadratic traction function involving the nominal stress ratios reaches the value one. This criterion can be represented as follows:

(12) $${\left\{\frac{\sigma_{n}}{\sigma_{n}^{0}}\right\}}^{2}\;+\;{\left\{\frac{\tau_{s}}{\tau_{s}^{0}}\right\}}^{2}\;+\;{\left\{\frac{\tau_{t}}{\tau_{t}^{0}}\right\}}^{2}\;=\;1$$

where *σ_n_* is the cohesive tensile strength; *τ_s_* and *τ_t_* are shear stresses of the interface; and *n*, *s*, and *t* refer to the direction of the stress component, as shown in Figure 5.

Modeling of damage allows for simulating both the degradation and failure of the bond between the two cohesive surfaces. The mechanism of failure includes the damage initiation criterion and a damage evolution law. The initial response is assumed to be linear elastic, and once a damage initiation criterion is met, damage can then occur according to a prior defined damage evolution law. In order to describe the damage evolution, the linear softening model is expressed in terms of fracture energy. The description of this model is available in the ABAQUS material library [38]. For describing the dependency of the fracture energy to the opening and sliding failure modes, the Benzaggah–Kenane (BK) and power law fracture criteria were used, which can be represented by:

(13) $${\left\{\frac{G_{n}}{G_{f}^{n}}\right\}}^{\eta }\;+\;{\left\{\frac{G_{s}}{G_{f}^{s}}\right\}}^{\eta }\;+\;{\left\{\frac{G_{t}}{G_{f}^{t}}\right\}}^{\eta }\;=\;1$$

In the above expression, the quantities *G_n_*, *G_s_*, and *G_t_* denote the work done by the interfacial stresses and its conjugate separation in the normal, the first, and the second shear directions, respectively; *η* is a cohesive property parameter; and *G_nf_*, *G_sf_*, and *G_tf_* represent the critical fracture energies required to cause failure in the normal, the first, and the second shear directions, respectively.

#### 2.2.2. Modeling of CCS Debonding

For modeling CCS failure, a separate part that represents the concrete cover was first created. Then, the interface between the concrete beam and the concrete cover part was modeled by considering a cohesive surface at the level of tension steel reinforcement. Eight-node 3D cohesive elements (element COH3D8 in ABAQUS) were used to model the interface layer based on a proper bond-slip model. The input values for the cohesive model in this interface were developed in this study. In order to find the values of initial stiffness, shear strength, and fracture energy that yield the best fit, simulations were performed, and the FE results were compared with experimental results of beams that were tested by Al-Saawani et al. [6]. The following relations for initial stiffness, *K_o_*, shear strength, *τ_max_*, and fracture energy, *G_cr_*, as a function of the concrete properties, were proposed:

(14) $$K_{o}\;=\;\frac{G_{c}}{t_{c}}$$

(15) $$\tau_{max}\;=\;f_{ct}$$

(16) $$G_{cr}\;=\;0.15{\left(f_{c}^{\prime }\right)}^{0.2}$$

where *t_c_* is the interface thickness, *G_c_* is the shear modulus of concrete in MPa, and *f_ct_* is the tensile strength of concrete in MPa calculated by Equation (9). The initiation of damage and failure mechanism were modeled considering the same approach used in the case of modeling IC debonding described earlier.

### 2.3. Model Geometry and Element Types

Three-dimensional simulations were performed to get an accurate approximation of the overall flexural behavior and type of debonding failure of the strengthened beams. The concrete was modeled using an eight-node reduced-integration linear brick element (C3D8R in ABAQUS). Steel reinforcements were modeled as embedded elements represented by two-node linear 3D truss elements (element T3D2 in ABAQUS). The truss elements have three degrees of freedom at each node, which include translations in the nodal *x*, *y*, and *z* directions. The truss element was defined by its cross-sectional area. The unidirectional CFRP reinforcement was represented by four-node reduced-integration shell element (element S4R in ABAQUS), because these elements consider the properties of orthotropic materials.

**Figure 6:**
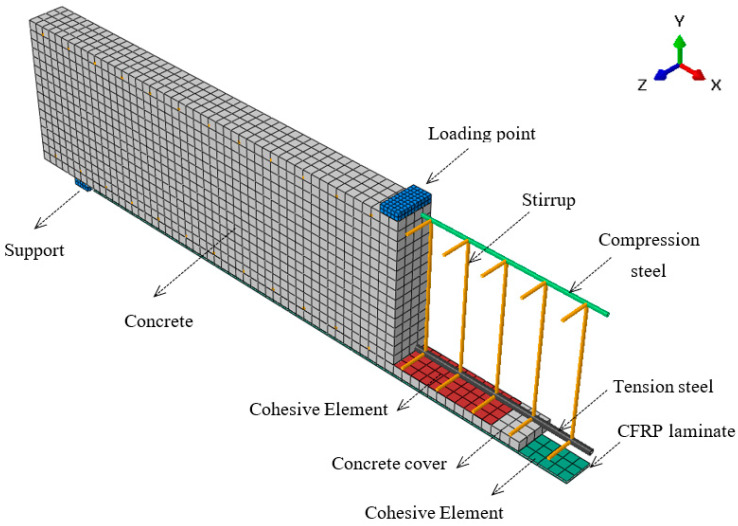
Geometry and elements used in the FE simulation.

In order to reduce the computational demand, the double symmetry of beam geometry and loading was utilized; thus, only a quarter of the strengthened beam was modeled upon applying appropriate boundary conditions. Figure 6 shows the three-dimensional model used, with the finite element mesh showing the geometry, different elements used, and applied load.

### 2.4. Mesh Size and Boundary Conditions

**Figure 7:**
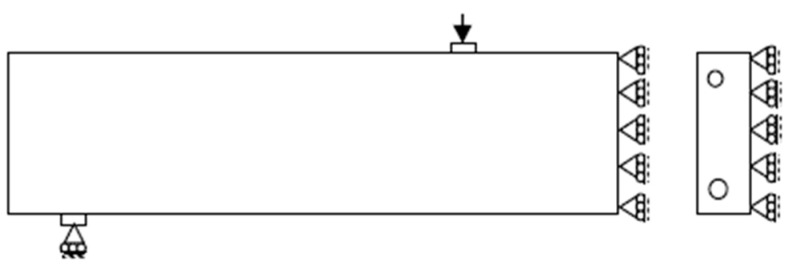
Symmetry boundary conditions used in the FE simulation.

For modeling concrete members, Bažant and Oh [44] recommended element sizes to be three times the size of the maximum coarse aggregate (3*d_a_*). In his study, different mesh sizes were used in the FE simulation in order to obtain convergence as well as numerical results that are in good agreement with experimental measurements. A moderately fine mesh of 25 mm element size was adopted in this study as it provided good accuracy and was computationally less demanding compared to the 12.5 mm element size that was tried. Boundary conditions that represent the supports and specify values of displacement and rotation variables at appropriate nodes were applied. The boundary conditions for a quarter of the beam are shown in Figure 7.

The element type in ABAQUS controls the element characteristics of the mesh. The mesh controls selected in the developed FE model are Hexahedron element meshing, while the type of mesh has been considered as structured. The reason for this selection of meshing type is because the geometry of elements used in modeling FRP-strengthened beams is not complex. In addition, the geometric order in ABAQUS controls the number of nodes on the selected elements. The selection between first-order or second-order elements is mostly a trade-off between computational expense and the accuracy of results. In addition, the class of problem being modeled dictates the proper choice of the element for FE modeling. For example, in case of modeling the contact with bending, the choice of first-order with reduced-integration elements is preferred, with a sufficient number throughout the thickness.

## 3. Model Calibration and Validation

**Figure 8:**
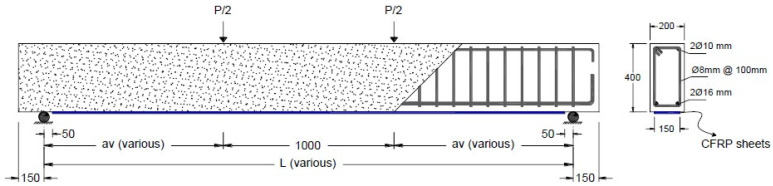
Geometry and reinforcement details of the analyzed beams [6].

To validate the developed numerical model, FE simulations were conducted for seven FRP-strengthened beams that were tested by Al-Saawani et al. [6]. These beams were chosen because of the detailed test results that are available and easily accessible, including load-deflection curves, FRP strains, interfacial shear stress between FRP and concrete, and cracking patterns. In addition, these beams have experienced both debonding failures, namely IC debonding at the middle of the beam or CCS failure at the plate-end region. The analyzed beams were constructed with different clear-span lengths, which gave a wide range of shear span-to-depth ratios of 1.5 to 7.0. Table 3 shows details of the beams and lists values of the shear span, *a_v_*, and the values of *a_v_*/*d_s_*. The cross-section configuration and details of the steel and CFRP reinforcements for such beams are shown in Figure 8. The mechanical properties of steel and CFRP reinforcements used in FE simulation are shown in Table 4 and Table 5, respectively.

**Figure 9:**
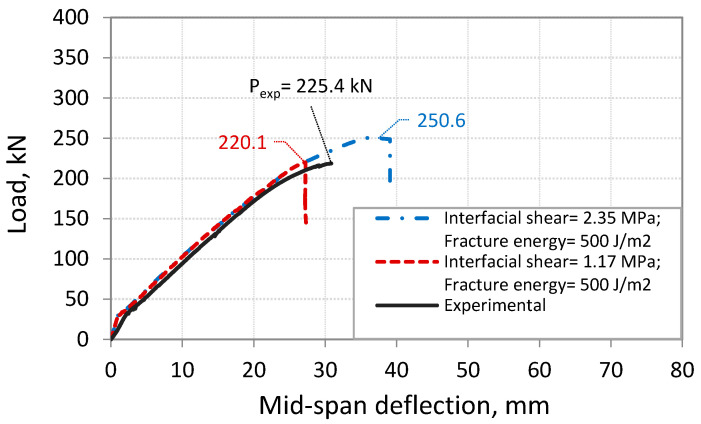
Comparison between experimental and FE results for different values of *τ_max_*.

**Figure 10:**
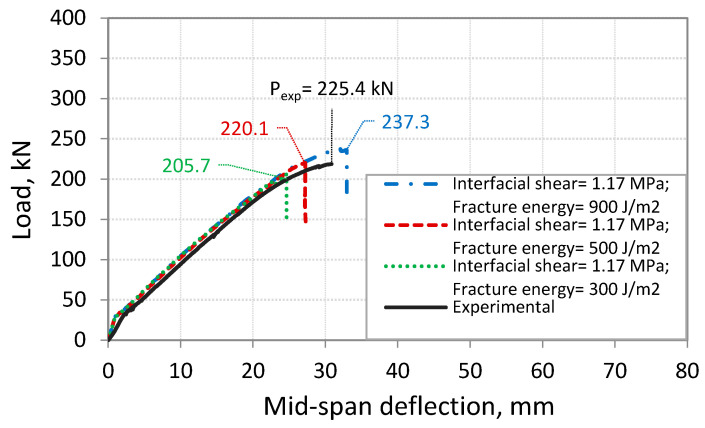
Comparison between experimental and FE results for different values of *G_cr_*.

This part presents the calibration of the developed FE model through running FE simulations for beam S-3.5, which has failed by IC debonding [6]. The results from the FE model show that the predicted capacity for beam S-3.5 at IC debonding failure was overestimated when using the interface properties suggested by Lu et al. [20]. The predicted capacity at IC debonding for the beam S-3.5 is found to be 11.6% higher than the experimental value. The cause for this difference in the load capacity is related to the estimation of the behavior of the interface between CFRP and concrete using the bond-slip model proposed by Lu et al. [20]. To improve the accuracy of the predicted capacity at IC debonding failure obtained from the FE model, a modification is suggested for the properties of the interface between CFRP and concrete. This modification is mainly related to the maximum shear stress, *τ_max_*, and the fracture energy, *G_cr_*. The maximum shear stress, *τ_max_*, calculated by Lu et al. [20] and shown in Table 2, provides an upper limit for *τ_max_* of 2.35 MPa for the analyzed beam S-3.5. The conducted FE analysis showed that this value is high because of the higher load capacity at IC debonding failure that was obtained considering Lu et al.’s model [20] compared to the test value, as shown in Figure 9. Therefore, different values of *τ_max_* were investigated, and a reduced value of 50% of that calculated gave better predictions of the load at IC debonding failure, as shown in Figure 9. This use of a reduced value of *τ_max_* was also observed by Sakr [37] to obtain better predictions. For the fracture energy, *G_cr_*, the value obtained using Lu et al.’s model [20] for the analyzed beam S-3.5 is 300 J/m^2^. This small value of *G_cr_* gives a lower capacity at IC debonding failure, as shown in Figure 10. In fact, previous research studies have indicated fracture energy values ranging from 300 to 1500 J/m^2^ [37,45,46]. To investigate the extent to which *G_cr_* can affect the FE results, numerical simulations were performed on the beam S-3.5 considering *G_cr_* values of 300, 500, and 900 J/m^2^. The simulations showed that *G_cr_* has an influence on the ultimate capacity at IC debonding failure, as seen in Figure 10. In this study, the value 500 J/m^2^ was selected, as it provided a load capacity at IC debonding that is in good agreement with the experimental results.

The input values for the bond-slip model that describes the CZM used to predict CCS failure were developed in this study. FE simulations were performed on the beam S-3.0, which experimentally failed by CCS [6], in order to find the values of initial stiffness, shear strength, and fracture energy that yield the best fit. As mentioned earlier in the model development section, the input values were suggested as a function of the concrete properties. Results from FE analysis were then compared with experimental results [6], which were in good agreement. The following subsections present details on the validation of the developed FE model by comparisons with the experimental results.

### 3.1. Ultimate Capacity and Mode of Failure

The results obtained from the FE simulations for seven FRP-strengthened beams [6] are listed in Table 6. The results include the ultimate capacities and the two critical debonding modes of failure, as obtained from the FE simulations for the tested beams. The comparisons presented in Table 6 show that the FE model was capable of predicting the type of debonding failure (i.e., IC debonding or CCS failure mode) for the experimental beams that had various values of shear span-to-depth ratio. The comparisons also show a good agreement in the ultimate capacities obtained from the FE analysis as compared to the experimental results for the FRP-strengthened beams. In general, the ratios of the experimental to numerical ultimate capacities (*P_u,exp_*/*P_u,FEM_*) were within 5%, except for the beams S-2.0 and S-1.5, for which the ratio of *P_u,exp_*/*P_u,FEM_* was 0.94 and 0.92, respectively. These comparisons indicate that the developed FE model is valid and can be used as a tool to predict the flexural behavior and strength at debonding failure for RC beams strengthened with FRP composites.

### 3.2. Load-Deflection Curves

**Figure 11:**
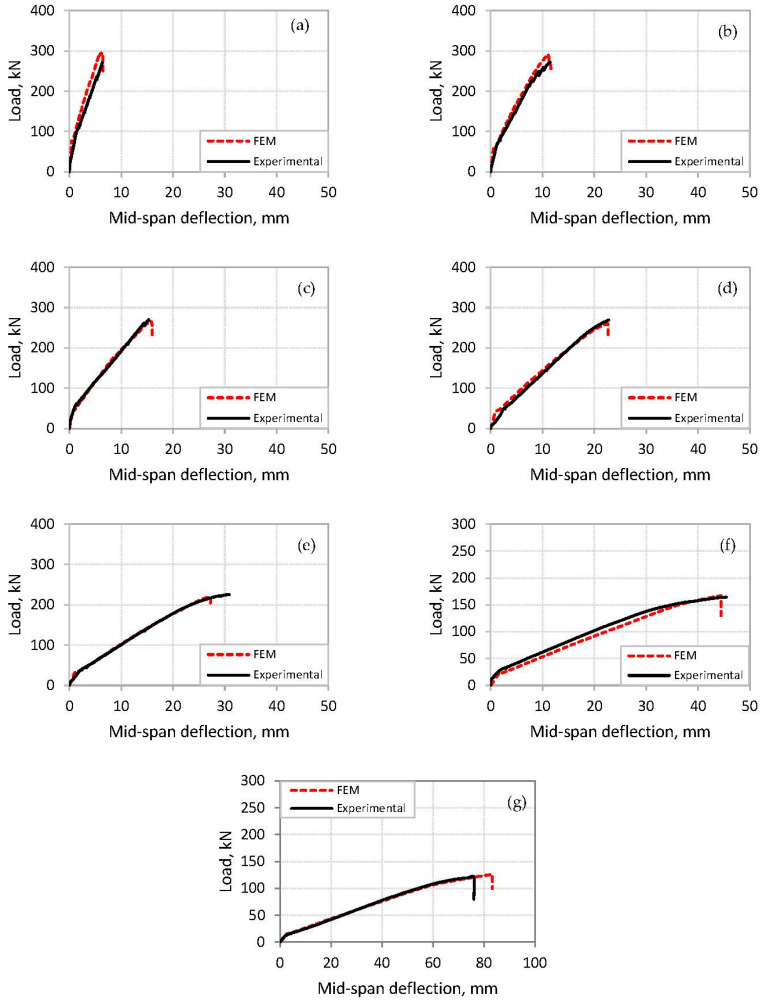
Load-deflection curves of analyzed beams obtained from experiments [6] and FE model: (**a**) beam S-1.5; (**b**) beam S-2.0; (**c**) beam S-2.5; (**d**) beam S-3.0; (**e**) beam S-3.5; (**f**) beam S-5.0; (**g**) beam S-7.0.

Comparisons of the load versus mid-span deflection curves of the beams tested by Al-Saawani et al. [6] and the FE analysis are shown in Figure 11. It is shown that the finite-element model can predict the behavior of the FRP-strengthened beams at cracking, yielding of tension steel, and IC debonding. In addition, the load at cracking, at yielding of tension steel, and at failure, along with the corresponding displacements, are in good agreement with the experimental results.

### 3.3. Strains in CFRP Reinforcement

**Figure 12:**
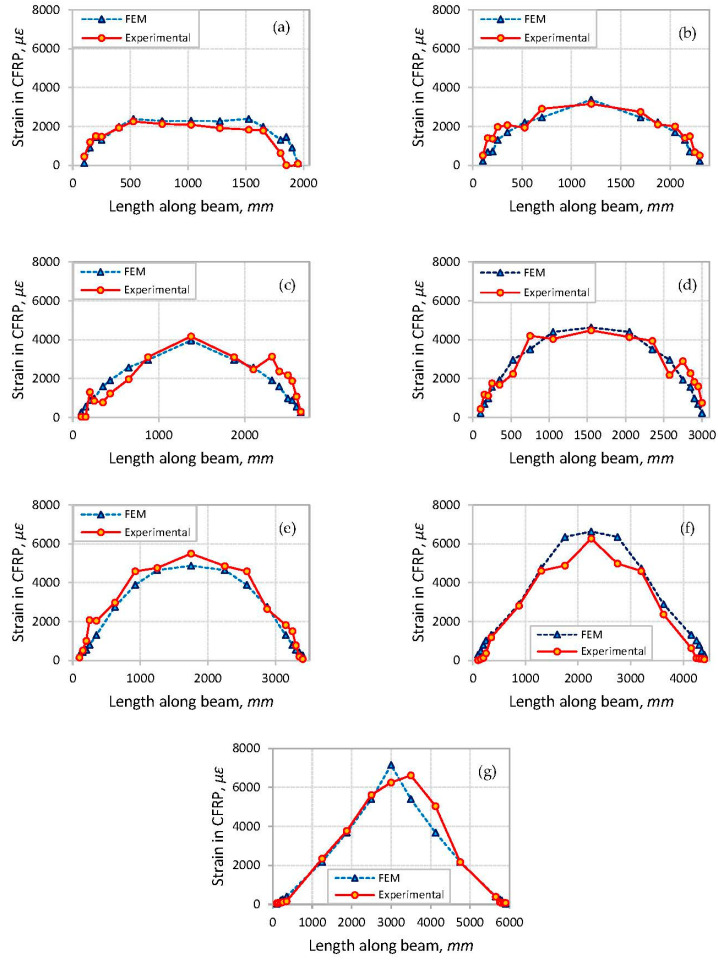
Comparison of strain distribution along the CFRP laminates from FE analysis and experimental results [47]: (**a**) beam S-1.5; (**b**) beam S-2.0; (**c**) beam S-2.5; (**d**) beam S-3.0; (**e**) beam S-3.5; (**f**) beam S-5.0; (**g**) beam S-7.0.

Comparisons of the axial tensile strain distribution along the CFRP laminate obtained from the FE analysis and the experimental study [47] were conducted and are shown in Figure 12. The predicted variations of strain along the CFRP laminates generally follow the bending moment variation along the beam length. In addition, the CFRP strains obtained from FE analysis are found to be quite similar to the measured strains obtained from the experimental results [47]. It can be seen from Figure 12 that the strain values increased linearly from the end of the CFRP laminates to the sections corresponding to the loading points and then stayed at a constant level within the constant-bending-moment region. This is particularly clear in the case of the beams with shear span-to-depth ratios ranging from 1.5 to 3.0, which failed by CCS at the plate-end region. In the case of beams with increased value of shear span-to-depth ratio, the strain values of the CFRP laminates were seen to further increase at the constant-moment region, with their highest values at the middle of the beam.

**Figure 13:**
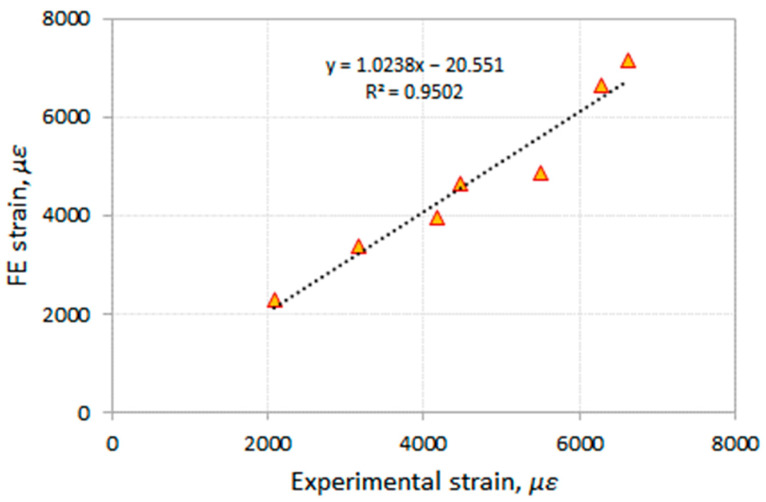
Comparisons of CFRP strains at the middle of the beam obtained from FE analysis and experimental results [47].

Comparisons of the CFRP strains at the middle of the beam obtained from the FE analysis and the experimental results [47] for the analyzed beams at debonding failures are shown in Figure 13. It can be seen from the figure that the CFRP strains obtained from the FE analysis are in good agreement with the strain values reported from the experimental study [47].

### 3.4. Crack Patterns

**Figure 14:**
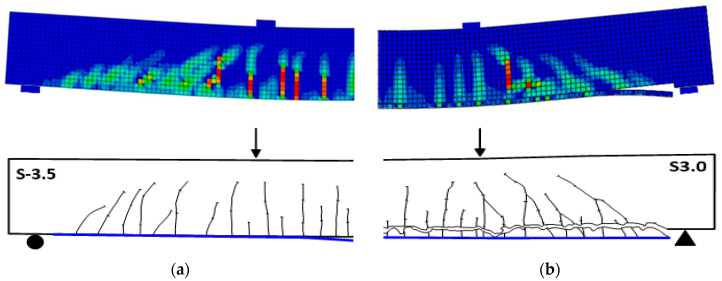
Comparison between plastic strain distribution from FE simulation and crack patterns from experimental results [6]: (**a**) beam S-3.5; (**b**) beam S-3.0.

The concrete damage plasticity model does not have a notation for the development of cracks at the material integration point. Therefore, it was assumed that cracking initiates at the points where the maximum principal plastic strain is positive, following Lubliner et al. [48]. Figure 14a shows a comparison between plastic strain distribution obtained from the FE analysis and crack patterns obtained from the experimental study [6] for the strengthened beam S-3.5, which failed by IC debonding. Similarly, Figure 14b shows the same comparison for the beam S-3.0, which failed by CCS [6]. It can be seen from the figure that the cracking patterns correspond well to the experimental crack distributions until failure. Both experimental and FE analysis show similar cracking pattern for both of the strengthened beams. In the experimental study [6], adequate steel stirrups were used in order to prevent the occurrence of shear failure for the strengthened beams. The spacing of stirrups had an important role in the spacing of the developed cracks. At the earlier stages of loading, the developed cracks were flexural cracks propagating in a vertical direction. With increased loading, more cracks developed at the shear span, which were mainly flexural-shear cracks that started at a vertical direction then propagated in an inclined direction to the beam longitudinal axis.

## 4. Parametric Study

### 4.1. Effect of Shear Span-to-Depth Ratio on Debonding Failure

**Figure 15:**
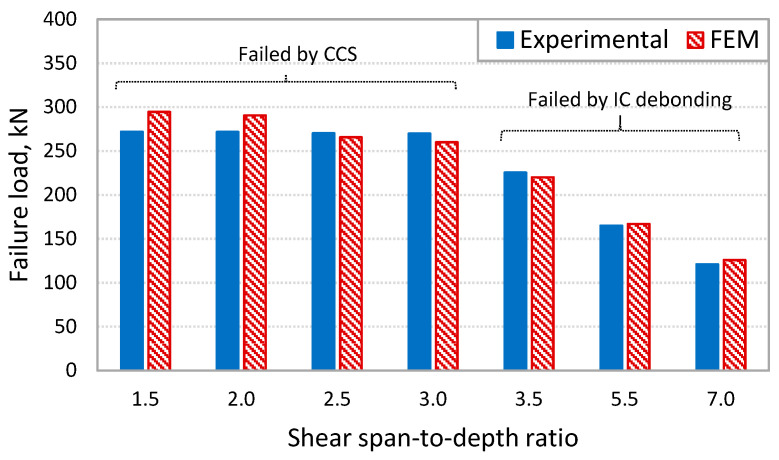
Comparison of FE analysis with experimental results [6] on the effect of *a_v_*/*d_s_* on debonding failures.

Shear span-to-depth ratio (*a_v_*/*d_s_*) is one of the main parameters that has an influence on the type of debonding failure in RC beams strengthened with externally bonded CFRP covering the entire span [6]. The developed FE model was used to simulate seven FRP-strengthened beams from the experimental study [6]. These beams had a wide range of av/ds from 1.5 to 7.0. Figure 15 shows the predicted capacities using the developed FE model for the beams with various values of *a_v_*/*d_s_* compared to the experimental values [6]. It can be seen from the figure that the results obtained from the numerical analysis confirm the experimental findings on the effect of *a_v_*/*d_s_* on shifting the failure mode from IC debonding (in the case of beams with *a_v_*/*d_s_* of 3.5 and higher) to CCS failure at the plate end in the case of beams with *a_v_*/*d_s_* values of 3.0 and lower.

### 4.2. Effect of Steel Stirrups on Debonding Failure

The developed FE model was used to investigate the effect of stirrup spacing on the debonding failure load for the two common debonding failures in FRP-strengthened beams. FE simulations were conducted for two beams: one prone to fail by plate-end debonding, and the other prone to fail by IC debonding. In particular, two FRP-strengthened beams with *a_v_*/*d_s_* values of 2.5 and 5.0 (beams S-2.5 and S-5.0) were chosen from the experimental study [6] in order to conduct the parametric study. For each beam, FE simulations were conducted where the variable was the spacing of stirrups: 100, 200, and 300 mm. These values represent a stirrup spacing to effective beam depth ratio of 0.28, 0.57, and 0.85, respectively. The diameter of stirrups of 8 mm was kept constant in all beams.

**Figure 16:**
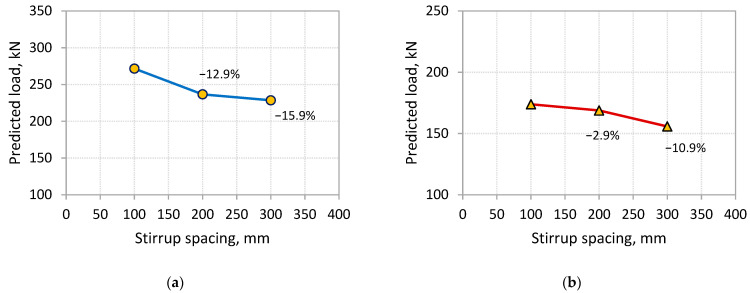
Effect of stirrup spacing on the capacity at debonding failures: (**a**) beams with *a_v_*/*d_s_* = 2.5; (**b**) beams with *a_v_*/*d_s_* = 5.0.

The results of the FE analysis are shown in Table 7 and Figure 16, which include the predicted failure load and corresponding mode of failure. The analysis results indicated that increasing the spacing of the steel stirrups causes a reduction of the load capacity for the strengthened beams in either mode of debonding failure (IC debonding and CCS failure). The reduction in load capacity was more pronounced in the case of beams that are predicted to fail by CCS at the FRP plate-end region. The percentage decrease in load capacity was 12.9% and 15.9% for the beams S-2.5/S-200 and S-2.5/S-300 with steel stirrups spaced at 200 and 300 mm, respectively, compared to the beam S-2.5/S-100 with stirrups spaced at 100 mm. In fact, the reduction in load capacities at CCS failure with increased spacing of stirrups is attributed to the reduced efficiency of stirrups in controlling the widening of shear cracks that occur at the shear span close to the plate end. Such poor control of the widening of the shear cracks at the plate-end region increased the horizontal and vertical components of the relative displacement associated with such cracks. This, in turn, caused an increase in the interfacial shear and normal stresses at the plate end, which triggered the CCS failure at decreased load capacities of the strengthened beams. These numerical findings agree with the experimental results reported in the recent study conducted by Al-Negheimish et al. [8] that reported reduced capacities of 11.6% and 15.9% in the tested beams that failed by CCS when the stirrup spacing increased from 100 mm to 150 mm and 250 mm, respectively.

In the case of beams that are prone to fail by IC debonding, the decrease in the load capacity was marginal in the case of the beam S-5.0/S-200 with stirrups spaced at 200 mm compared to the beam S-5.0/S-100 with stirrups spaced at 100 mm. This is because the IC debonding in these two beams initiated at the middle of the beam, close to the point load as induced by flexural cracks. In this area, the vertical stirrups have a limited role in restricting the widening of the flexural cracks, and this explains the marginal change in load capacity at IC debonding, which was basically induced by such flexural cracks in the middle of the strengthened beam.

**Figure 17:**
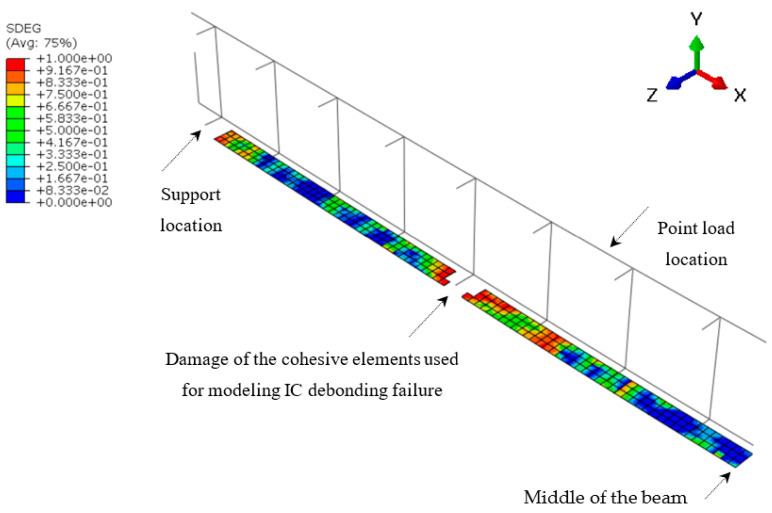
Failure of the beam S-5.0/S-300 used in the parametric study.

In contrast, a decrease in the load capacity of 10.4% was observed in case of the analyzed beam S-5.0/S-300 with steel stirrups spaced at 300 mm when compared to the beam S-5.0/S-100 with stirrups spaced at 100 mm. This is attributed to the occurrence of IC debonding at the shear span due to flexural-shear cracks rather than flexural cracks at the middle of the beam. Figure 17 shows the location of IC debonding failure in the beam S-5.0/S-300, which was initiated at a distance of 550 mm measured from the point load towards the shear span. The failure by IC debonding was evidenced by the damage of the cohesive elements that were used in the FE model to represent the IC debonding failure. This was checked by the maximum value of the quadratic nominal stress damage initiation (QUADSCRT) criterion and the overall scalar stiffness degradation (SDEG) variable in ABAQUS, which indicate that the initiation criterion and damage has been satisfied (as shown in Figure 17). Generally, the cracks at the shear span are inclined, and thus the steel stirrups have an important role in restricting the widening of such cracks. Therefore, increasing the spacing of stirrups to 300 mm (representing 85% of the effective depth of the beam S-5.0/S-300) reduced the effectiveness of stirrups in the shear span, and thus caused more widening of the cracks. This eventually triggered the failure by IC debonding at lower load capacity of the strengthened beam. It is worth noting that many design codes limit the maximum spacing of stirrups to the range of 0.5 to 0.7 the effective depth of the concrete beam. Therefore, within the allowable spacing of steel stirrups specified by design codes, the capacity at IC debonding failure should not affected by the spacing of steel stirrups.

**Figure 18:**
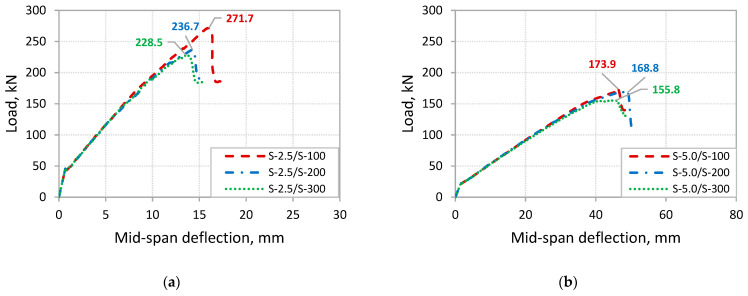
Effect comparison of load-deflection curves for the beams with various stirrup spacings: (**a**) beams with *a_v_*/*d_s_* = 2.5; (**b**) beams with *a_v_*/*d_s_* = 5.0.

Comparisons of the load versus mid-span deflection curves of the analyzed beams using the FE model are shown in Figure 18a, in the case of FRP-strengthened beams with av/ds of 2.5, and Figure 18b, for the beams with av/ds of 5.0. The plots show no effect of the stirrup spacing on the flexural stiffness of the FRP-strengthened beams for the two values of av/ds. However, a decrease in the flexural stiffness was observed at the segment of the load-deflection curves before the IC debonding failure for the beams with increased stirrup spacing of 200 and 300 mm as compared to the beam with steel stirrups spaced at 100 mm.

## 5. Conclusions

This paper presented the development of a finite-element model for simulating the two critical debonding failures in FRP-strengthened beams using the commercial software ABAQUS. The developed FE model used a concrete damaged plasticity model for concrete, an elastic perfectly plastic model for steel reinforcement, and a fracture-energy-based cohesive model to represent the interfaces related to the two critical debonding failures. The main findings of this investigation can be summarized as follows:

1. The developed FE model in this study simulates the flexural behavior and predicts the critical type of debonding failure (IC debonding and CCS failure) and the corresponding capacity at failure for the FRP-strengthened beam.
2. The FE results were validated by comparisons with the experimental results for FRP-strengthened beams having a wide range of shear span-to-depth ratios. The comparisons have confirmed the capability of the developed FE model to distinguish between the two critical debonding failure modes in FRP-strengthened beams and to predict the failure loads in close agreement with the experimental values.
3. A parametric study was conducted using the developed FE model in order to investigate the effect of shear span-to-depth ratio and the spacing of steel stirrups on the debonding failures in FRP-strengthened beams. The FE analysis showed the effect of shear span-to-depth ratio on the type of debonding failure for RC beams strengthened with CFRP laminates covering the entire span to the supports.
4. The results of the FE analysis showed that increasing the spacing of stirrups causes a reduction in the load-carrying capacity at CCS failure. In contrast, a marginal reduction in the load-carrying capacity in the case of the beams that fail by IC debonding was found if the allowable spacing of stirrups specified by design codes is not exceeded. However, increasing the stirrup spacing beyond the codes’ limit causes a change in the location of the IC debonding from the middle of the beam (induced by flexural cracks) to the shear span initiated by flexural-shear cracks at a reduced load-carrying capacity.

## Figures and Tables

**Table 1:** Values of plastic damage parameters used for concrete model.

Parameter	Dilation Angle (*Ψ*)	Eccentricity (*ϵ*)	*f_b_*_0_ */f_c_*_0_	*K*	Viscosity Parameter
**Value**	30°	0.1	1.16	0.667	0.0001

**Table 2:** Parameters of bond-slip model used in the FE study.

Parameter	*K*_0_ (MPa/mm)	*τ_max_* (MPa)	*G_cr_* (N/mm)	*β_w_*
**Value**	$\tau_{max}/\delta_{0}$ $\delta_{0}\;=\;0.0195\;\beta_{w}f_{ct}$	$1.5\;\beta_{w}f_{ct}$	$0.308\;\beta_{w}^{2}\sqrt{f_{ct}}$	$\sqrt{\frac{2.25\;-\;\frac{b_{f}}{b}}{1.25\;+\;\frac{b_{f}}{b}}}$

**Table 3:** Details of the simulated beams that were tested by Al-Saawani et al. [6].

Beam	Clear Span, *L* (mm)	Shear Span, *a_v_* (mm)	Shear Span-to-Depth Ratio, *a_v_*/*d_s_*
S-1.5	2050	525	1.5
S-2.0	2400	700	2.0
S-2.5	2750	875	2.5
S-3.0	3100	1050	3.0
S-3.5	3500	1250	3.5
S-5.0	4500	1750	5.0
S-7.0	6000	2500	7.0

**Table 4:** Properties of steel reinforcement used in FE simulation, tested by Al-Saawani et al. [6].

Property	Elastic Modulus (GPa)	Poisson’s Ratio	Yield Stress (MPa)
Tension steel	205	0.3	550
Compression steel	200		500
Stirrups	200		350

**Table 5:** Properties of the CFRP reinforcement used in FE simulation, tested by Al-Saawani et al. [6].

Parameter	*E*_1_ (GPa)	*E*_2_ (GPa)	*ν*_12_	*G*_12_ (GPa)	*G*_13_ (GPa)	*G*_23_ (GPa)	*f_u_* (MPa)
**Value**	95.8	6.143	0.29	1.55	1.22	1.22	984.6

**Table 6:** Ultimate capacities and failure modes obtained by experiments [6] and FE model.

Beam	Experimental Results			FE Analysis Results			*P_u,exp_*/*P_u,FEM_*	𝛿*_u,exp_*/𝛿*_u,FEM_*
	*P_u,exp_* (kN)	𝛿*_u,exp_* (mm)	Mode of Failure *	*P_u,FEM_* (kN)	𝛿*_u,FEM_* (mm)	Mode of Failure *		
S-1.5	271.9	6.4	CCS	294.5	6.2	CCS	0.92	1.03
S-2.0	271.7	11.4	CCS	290.5	10.9	CCS	0.94	1.05
S-2.5	270.2	15.3	CCS	265.7	15.7	CCS	1.02	0.97
S-3.0	269.8	22.8	CCS	260.0	22.5	CCS	1.04	1.01
S-3.5	225.4	30.9	ICD	220.1	27.2	ICD	1.02	1.14
S-5.0	165.1	45.4	ICD	167.0	44.5	ICD	0.99	1.02
S-7.0	121.0	76.0	ICD	125.7	83.2	ICD	0.96	0.91

* CCS = concrete cover separation failure; ICD = intermediate-crack-induced debonding failure.

**Table 7:** Ultimate capacities and failure modes obtained by FE analysis on the effect of stirrup spacing.

Beam	Shear Span-to-Depth Ratio	*S_v_* (mm)	*P_u,FEM_* (kN)	Δ*P_u,FEM_* (kN)	Mode of Failure *
S-2.5/S-100	2.5	100	271.7	-	CCS
S-2.5/S-200		200	236.7	−12.9%	CCS
S-2.5/S-300		300	228.5	−15.9%	CCS
S-5.0/S-100	5.0	100	173.9	-	ICD
S-5.0/S-200		200	168.8	−2.9%	ICD
S-5.0/S-300		300	155.8	−10.4%	ICD

* CCS = concrete cover separation failure; ICD = intermediate-crack-induced debonding failure.

## Data Availability

Data from this study can be made available upon request.
